# Power and sample size calculation for non-inferiority trials with treatment switching in intention-to-treat analysis comparing RMSTs

**DOI:** 10.1186/s12874-025-02604-3

**Published:** 2025-06-07

**Authors:** Austin Shih, Chih-Yuan Hsu, Yu Shyr

**Affiliations:** 1 Department of Mathematics, Vanderbilt University, Nashville, TN 37240 USA; 2 Department of Biostatistics, Vanderbilt University Medical Center, Nashville, TN 37203 USA; 3 Center for Quantitative Sciences, Vanderbilt University Medical Center, Nashville, TN 37203 USA

**Keywords:** Crossover, Intention-to-treat analysis, Non-inferiority trials, Restricted mean survival time

## Abstract

**Background:**

Difference in Restricted Mean Survival Time (DRMST) has attracted attention and is increasingly used in non-inferiority (NI) trials because of its superior power in detecting treatment effects compared to hazard ratio. However, when treatment switching (also known as crossover) occurs, the widely used intention-to-treat (ITT) analysis can underpower or overpower NI trials.

**Methods:**

We propose a simulation-based approach, named *nifts,* to calculate powers and determine the necessary sample size to achieve a desired power for non-inferiority trials that allow treatment switching, in ITT analysis using DRMST.

**Results:**

The *nifts* approach offers three options for a non-inferiority margin, assumes three entry patterns and generalized gamma distributions for event time, incorporates two distributions for dropout censoring, and provides five distribution options for switching. Real-world and simulated examples are used to illustrate the proposed method and examine how switching probability, switching time, the relative effectiveness of treatments, allocation ratio, entry patterns, and event time distribution influence powers and sample sizes. *nifts* adjusts the non-inferiority margins intended for NI trials without treatment switching to accommodate the presence of treatment switching in the designs. With the adjusted margins, the type I errors are well-controlled. The ratios of sample sizes with treatment switching to those without switching are close to 1, indicating no significant change in power at sample sizes without switching when using adjusted margins. The performance on power and sample sizes is not sensitive to the choice of switch time distributions.

**Conclusions:**

This simulation-based approach provides power and sample size calculation in NI trials with treatment switching, when comparing the RMSTs of two treatment groups in ITT analysis. With its comprehensive parameter settings, *nifts* will be useful for designing NI trials that allow for treatment switching. *nifts* is freely available at https://github.com/cyhsuTN/nifts.

**Supplementary Information:**

The online version contains supplementary material available at 10.1186/s12874-025-02604-3.

## Introduction

A randomized controlled trial (RCT) is regarded as the gold standard for assessing the effectiveness of new treatments. Among the various types of RCTs, a non-inferiority (NI) trial aims to demonstrate that a new treatment is not significantly worse than an existing one, while potentially offering additional benefits such as fewer side effects or lower costs. One increasingly popular approach for evaluating treatment effects in NI trials with time-to-event outcomes is to compare restricted mean survival times (RMST) between treatment groups [1–3]. RMST provides a straightforward summary by averaging survival times up to a specified time point [4] and does not rely on the proportional hazards (PH) assumption, which is frequently violated in clinical trials [5]. As a result, Royston and Parmar [6] suggested using the difference in RMSTs (DRMST) between treatment groups as an alternative to the hazard ratio (HR) for designing randomized trials with time-to-event outcomes, including power and sample size calculations. Furthermore, DRMST has greater power in detecting treatment effects compared to HR, even under the PH assumption [7, 8]. Several methods for determining powers and sample sizes in NI trials have been proposed. Weir and Trinquart [9] utilized DRMST, assuming Weibull survival distributions, uniform entry, and no-dropout censoring. Phadnis and Mayo [10] considered generalized gamma survival distributions and flexible entry and dropout censoring patterns, based on the Proportion Time (PT) assumption instead of the PH assumption. Other methods using the logrank test in NI trials include Jung et al. [11] and Barthel et al. [12], with a margin expressed on the hazard ratio scale. The former assumed exponential survival distributions with uniform entry during the accrual period. The latter considered a more complex setting, allowing for multi-arm survival trials, non-uniform accrual, non-proportional hazards, loss to follow-up and cross-over.

In RCTs, including NI trials, treatment switching from the therapy regimen of active control group to that of new experimental group may occur due to ethical concerns or other reasons [13, 14]. This switch may happen when a disease progresses, when healthcare providers believe the patient’s prognosis will improve with the experimental treatment, or when patients prefer the new treatment due to perceived benefits such as fewer side effects or greater convenience [13, 15]. However, treatment switching can confound the results of intention-to-treat (ITT) analysis, making it difficult to determine the true treatment effect. ITT analysis includes all participants with randomization and compares their responses to determine the treatment effect according to the initially assigned treatment groups, regardless of what treatment they received. This may potentially lead to underpowered trials and inconclusive results [14]. An alternative approach is per-protocol analysis that censors participants who switch treatments from the analysis. Nevertheless, this can heavily bias the results if there is a significant difference in prognosis between the included and excluded participants, particularly if the treatment switching is associated with prognostic variables [16]. Therefore, ITT analysis is still often used in the final analysis. Deng et al. [17] proposed a simulation-based approach to preview power reduction and sample sizes required in superiority trials with treatment switching in ITT analysis using the logrank test.

In this study, we propose a simulation-based approach, named *nifts,* to determine power and sample size in NI trials that involve treatment switching when comparing RMSTs between two treatment groups in ITT analysis. *nifts* adjusts the non-inferiority margins intended for NI trials without treatment switching to accommodate the presence of treatment switching in the designs. The approach offers three options for a non-inferiority margin, assumes three entry patterns and generalized gamma distributions for event times, incorporates two distributions for dropout censoring, and provides five distribution options for switching. To accelerate the computation of sample sizes, a monotonic smoothing technique is employed to estimate the power trend as sample sizes increase [18]. We utilize both real-world and simulated examples to illustrate the proposed method and examine how switching probability, switching time, the relative effectiveness of treatments, allocation ratio, entry patterns, and event time distribution influence power and sample sizes. *nifts* is freely available at https://github.com/cyhsuTN/nifts.

## Methods

### Non-inferiority trials using DRMST

**Fig. 1:**
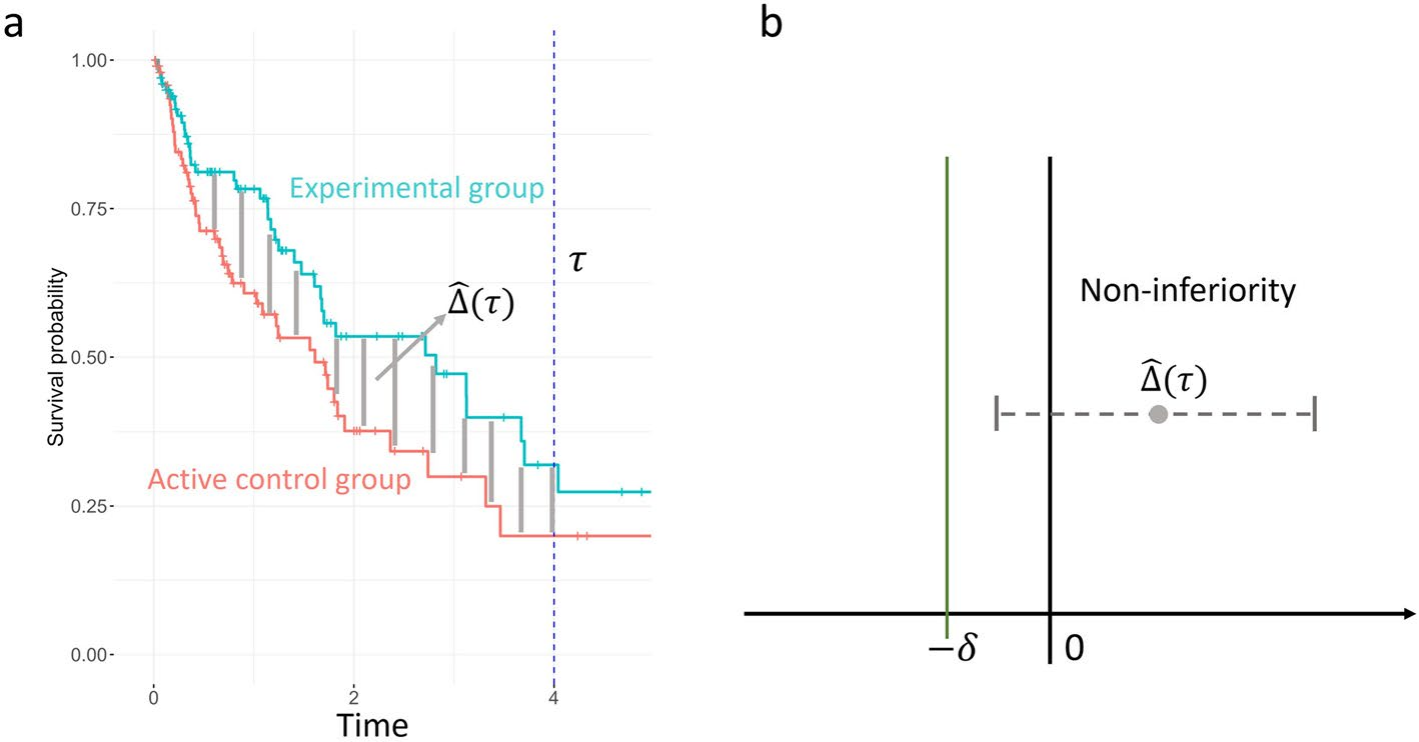
A non-inferiority test using DRMST. **a** Difference in RMSTs between two treatment groups. **b**) Non-inferiority holds if the lower bound of DRMST is larger than $-\delta$

Denote the survival functions for the active control group and the experimental group by ${S}_{1}(t)$ and ${S}_{2}(t)$, respectively. The restricted mean survival times (RMST) at a specified time $\tau \left(\tau>0\right)$ for the two groups are defined as ${R}_{i}\left(\tau \right)={\int }_{0}^{\tau }{S}_{i}(t) dt$,$i$= 1 and 2. The difference in RMSTs between the two groups (DRMST) is given by $\Delta \left(\tau \right)={R}_{2}\left(\tau \right)-{R}_{1}(\tau )$. The estimate for $\Delta \left(\tau \right)$ is $\widehat{\Delta }\left(\tau \right)={\widehat{R}}_{2}\left(\tau \right)-{\widehat{R}}_{1}(\tau )$, where ${\widehat{R}}_{i}\left(\tau \right)={\int }_{0}^{\tau }{\widehat{S}}_{i}(t) dt$ and ${\widehat{S}}_{i}(t)$ is the Kaplan–Meier (KM) estimate for ${S}_{i}(t)$. The aim of a non-inferiority trial using DRMST is to test ${H}_{0}:\Delta \left(\tau \right)\le -\delta$ vs ${H}_{1}:\Delta \left(\tau \right)>-\delta$, where $\delta>0$ is a prespecified margin.

$$\widehat{\Delta }\left(\tau \right)-{z}_{1-\alpha }\text{SE}\left(\widehat{\Delta }\left(\tau \right)\right)>-\delta,$$

if we reject the null hypothesis at a one-sided significance level of $\alpha$ and claim that non-inferiority holds, meaning the experimental treatment is not significantly worse than the active control (Fig. 1). Here, ${z}_{1-\alpha }$ represents the ($1-\alpha$)th quantile of the standard normal distribution, and $\text{SE}\left(\widehat{\Delta }\left(\tau \right)\right)=\sqrt{\widehat{Var\left({\widehat{R}}_{1}\left(\tau \right)\right)}+\widehat{Var\left({\widehat{R}}_{2}\left(\tau \right)\right)}}$. $\widehat{Var\left({\widehat{R}}_{i}\left(\tau \right)\right)}$ is the estimate for the variance of ${\widehat{R}}_{i}\left(\tau \right)$, whose explicit expression can be found in [9]. Both ${\widehat{R}}_{i}\left(\tau \right)$ and $\widehat{Var\left({\widehat{R}}_{i}\left(\tau \right)\right)}$ can be calculated using the *survfit* function in the *survival* R package. In addition, using flexible parametric models under the PH assumption for the estimation of DRMST can result in increased power when compared to the non-parametric KM estimation [8]. However, the PH assumption may be violated, given that this study involves treatment switching.

### The choice of margins

In this study, we propose three options for determining margins.

#### Preserved fraction of the RMST of the active control group

We aim for ${R}_{2}(\tau )$ to maintain at least the preserved fraction, ${f}_{1}$, of the RMST of the active control group, where $0<{f}_{1}<1$. The preserved fraction ${f}_{1}$ refers to the proportion of the active control group’s RMST that the experimental group’s RMST is considered acceptable to maintain. This can be expressed as ${R}_{2}\left(\tau \right)>{f}_{1}{R}_{1}\left(\tau \right)$. Thus, $\Delta \left(\tau \right)>-(1-{f}_{1}){R}_{1}(\tau )$ and $\delta =(1-{f}_{1}){R}_{1}(\tau )$.

#### Preserved fraction of the DRMST between the active control and hypothetical placebo groups

In this option, we aim for the RMST of the experimental group to be better than the RMST of the hypothetical placebo group, and the DRMST between the experimental and hypothetical placebo groups to maintain at least the preserved fraction, ${f}_{2}$, of the DRMST between the active control and hypothetical placebo groups, where $0<{f}_{2}<1$. The preserved fraction ${f}_{2}$ represents the proportion of the DRMST between the active control and hypothetical placebo groups that the DRMST between the experimental and hypothetical placebo groups is considered acceptable to maintain. This relationship can be expressed as ${R}_{2}\left(\tau \right)-{R}_{0}\left(\tau \right)>0$ and ${R}_{2}\left(\tau \right)-{R}_{0}(\tau )>{f}_{2}\left({R}_{1}\left(\tau \right)-{R}_{0}\left(\tau \right)\right)$, where ${R}_{0}\left(\tau \right)={\int }_{0}^{\tau }{S}_{0}(t) dt$ and ${S}_{0}(t)$ is the survival function for the hypothetical placebo group. Typically, ${R}_{1}\left(\tau \right)-{R}_{0}\left(\tau \right)>0$ holds, so $\delta =\left(1-{f}_{2}\right)\left({R}_{1}\left(\tau \right)-{R}_{0}\left(\tau \right)\right)$.

#### Conversion from the hazard ratio

Given ${S}_{1}(t)$ and assuming proportional hazards, a margin ($1/\theta$) for the hazard ratio (HR) of the experimental group to the active control group can be converted to a margin for DRMST from ${\text{HR}}_{21} < 1/\theta$ to $\Delta \left(\tau \right)>-\delta$, where $\delta ={R}_{1}\left(\tau \right)-{R}_{\theta }\left(\tau \right)$ with ${R}_{\theta }\left(\tau \right)={\int }_{0}^{\tau }{\left({S}_{1}\left(t\right)\right)}^{1/\theta } dt$ and $0<\theta <1$.

### The design setting and assumption

**Fig. 2:**
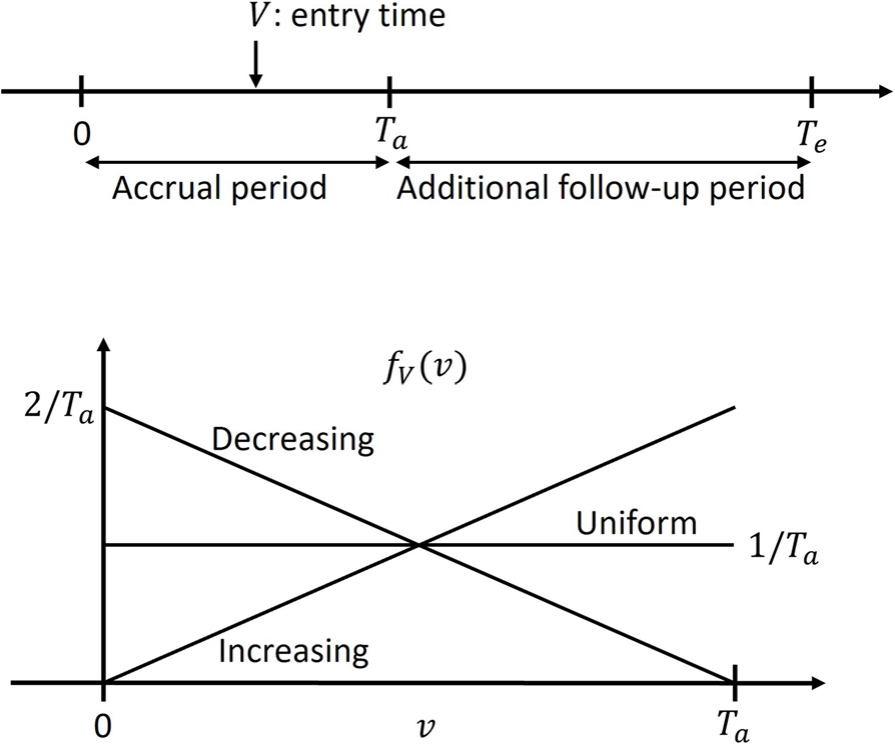
Accrual time and trial duration, along with three entry patterns

Denote the trial duration by ${T}_{e}>0$ and the accrual time during which participants are recruited by ${T}_{a}\ge 0$, where ${T}_{e}$ and ${T}_{a}$ are pre-specified constants. ${T}_{e}-{T}_{a}\ge 0$ is the additional follow-up time. If ${T}_{a}=0$, all participants are assumed to enter the study at its start. Otherwise, participants are assumed to enter the study with one of the three patterns during the accrual period: decreasing entry, uniform entry, and increasing entry. Specifically, the density function of the entry time $V$ is expressed as:

$$f_V\left(v\right)=a^\ast+b^\ast v,0\leq v\leq T_{a,}$$

where ${a}^{*}=2/{T}_{a}$ and ${b}^{*}=-2/{T}_{a}^{2}$ for decreasing entry; ${a}^{*}=1/{T}_{a}$ and ${b}^{*}=0$ for uniform entry; ${a}^{*}=0$ and ${b}^{*}=2/{T}_{a}^{2}$ for increasing entry (Fig. 2). The choice of entry patterns depends on participants’ accrual. We assume participants are randomly assigned to the active control group or the experimental group with an allocation ratio of $r$, where $r$ is defined as the ratio of the participants in the experimental group to those in the active control group.

Denote the survival times for participants in the active control and experimental groups by ${T}_{1}$ and ${T}_{2}$, respectively. ${T}_{1}$ and ${T}_{2}$ are random variables and differ for each participant. We assume that ${T}_{1}$ and ${T}_{2}$ follow generalized gamma distributions [19]:

$${f}_{T}\left(t|a, b, k\right)=\frac{b}{\Gamma \left(k\right)}\frac{{t}^{bk-1}}{{a}^{bk}} {e}^{-{\left(\frac{t}{a}\right)}^{b}}, t>0,$$

with the same shape parameters of $b>0$ and $k>0$ but different scale parameters of $a>0$. If $b=1$, the generalized gamma distribution reduces to the gamma distribution. If $k=1$, it reduces to the Weibull distribution. If $b=1$ and $k=1$, it simplifies the exponential distribution. When the three-parameter generalized gamma distribution reduces to two-parameter gamma or Weibull distributions, the scale and shape parameters can be easily determined by a given median survival of $m$ and a survival probability at a specific time $t$. For example, the scale and shape parameters of the Weibull distribution in the active control group are obtained by solving the equations: $\text{a} {\left(\text{log}2\right)}^{1/\text{b}}= {m}_{1}$ and $\text{exp}(-{\left(t/\text{a}\right)}^{\text{b}})$ = survival probability.

Denote the censoring times for participants in the control and experimental groups by ${C}_{1}$ and ${C}_{2}$, defined as the duration from randomization to either dropping out of the trial or reaching the end of the trial if participants don’t experience the event of interest. Therefore, the censoring time comprises dropout censoring and administrative censoring, and its distributions given $V=v$ can be formulated as follows [20]:

$$f\left(c|v\right)=d\left(c\right)I\left(0<c<{T}_{e}-v\right)+ \overline{D }\left({T}_{e}-v\right)I\left(c={T}_{e}-v\right),$$

where $d\left(c\right)$ and $\overline{D }\left(c\right)$ are the density function and survival function of the dropout censoring, respectively. $I(\bullet )$ is the indicator function. The dropout censoring is assumed to follow either a uniform distribution or an exponential distribution, where the parameter is determined by a given censoring probability of the active control group under no treatment switching (see Supplementary Materials for details). If a high proportion of patients dropped out in the early stages according to previous studies, selecting an exponential dropout censoring distribution is more appropriate. For the scenario of no dropout censoring, we set $P\left(c={T}_{e}-v|v\right)=1$. Additionally, the distributions of the censoring times in the two groups are assumed to be the same.

### Treatment switching

During the design stage, *nifts* anticipates the possibility that participants assigned to the active control group may switch their therapy regimen to that of the experimental group if certain predetermined conditions are met. For example, if patients with cancer have a disease progression before death (assume death is the event of interest), they may switch from the standard treatment to the new treatment after disease progression and evaluation by the investigators [13]. It is important to note that treatment switching being considered here is expected by design and not due to (1) a mid-study design change (e.g., after a decision that the experimental arm has strong evidence of benefit) or (2) non-compliance with control treatment. Denote the switching time by $S$, defined as the duration from randomization to the moment when a participant may switch, with a probability ${p}_{s}$. The switching probability ${p}_{s}$ is the likelihood that a participant who qualifies for treatment switching will change their treatment from the designated group to the other group after evaluation by healthcare professionals.

Five options for the distributions of the switching time are provided (Table 1), as used in [17]. The first three options assume $S$ is correlated with ${T}_{1}$, while the other two options assume $S$ is not correlated with ${T}_{1}$. The distributions *s.dist* = “unif” or “beta” are considered if the switching time always occurs before the event time. In contrast, *s.dist* = “gamma” or *s.dist* = “indepExp” is used if the switching time does not always occur before the event time. Furthermore, we consider *s.dist* = “gamma” if the switching time may be associated with the event time, and *s.dist* = “indepExp” otherwise. The parameters in the assumed distributions are determined based on the given values of ${r}_{s}$ and ${\rho }_{s}$ (See Supplementary Materials for details). ${r}_{s}=E(S)/E\left({T}_{1}\right)$ denotes the ratio of the average switching time to the average survival time of the active control group, and ${\rho }_{s}$ denotes the correlation between $S$ and ${T}_{1}$. If inputting ${r}_{s}$ and ${\rho }_{s}$ is challenging, trying different values can help obtain conservative power and sample sizes.

**Table 1:** Five options for the switching time distribution are provided

	Options	Property
$S$ is correlated with ${T}_{1}$. Assume $S = X{T}_{1}$ and $X$ is independent of ${T}_{1}$	*s.dist* = “unif”:$X \sim U(\text{0,1})$	$S<{T}_{1}$
	*s.dist* = “beta”:$X \sim \text{Beta}({\text{shape}}_{1}=a,{\text{shape}}_{2}=b)$, ${r}_{s}=a/(a+b)$, and${\rho }_{s}=\left\{\frac{a}{a+b}\sqrt{Var\left({T}_{1}\right)}\right\}/ {\left\{{\left(\frac{a}{a+b}\right)}^{2}Var\left({T}_{1}\right)+\frac{ab}{{\left(a+b\right)}^{2}\left(a+b+1\right)}E\left({T}_{1}^{2}\right)\right\}}^{1/2}$	$S<{T}_{1}$
	*s.dist* = “gamma”:$X \sim \text{Gamma}(\text{shape}=a,\text{rate}=b)$, ${r}_{s}=a/b$, and ${\rho }_{s}=\left\{\frac{a}{b}\sqrt{Var\left({T}_{1}\right)}\right\}/ {\left\{{\left(\frac{a}{b}\right)}^{2}Var\left({T}_{1}\right)+\frac{a}{{b}^{2}}E\left({T}_{1}^{2}\right)\right\}}^{1/2}$	
$S$ is not correlated with ${T}_{1}$	*s.dist* = “indepExp”:$S \sim \text{Exponential}(\text{rate}=b)$, $b={\left({r}_{s} E\left({T}_{1}\right)\right)}^{-1}$	
	*s.dist* = a numeric value:$S$ is a specific time, e.g., $S=0$ denotes the switch occurs at the start of the study	

**Fig. 3:**
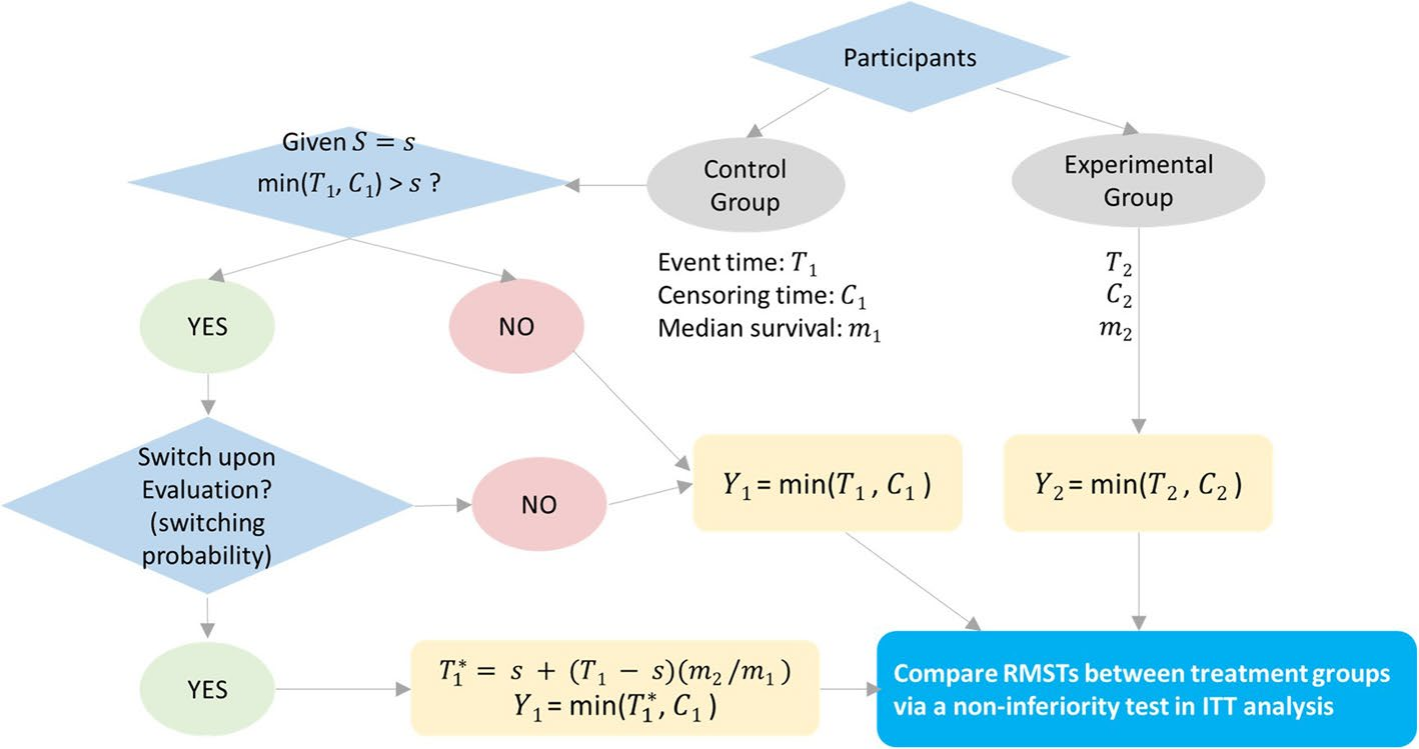
A flowchart illustrating non-inferiority trials that allow treatment switching. Based on this, *nifts* calculates power and required sample sizes

The survival time for participants starting from switching is assumed to increase by ${m}_{2}/{m}_{1}$, based on the rank preserving structural failure time model (RPSFTM) [21]. Thus, the survival time of the participants with treatment switching will be ${T}_{1}^{*} = s + ({T}_{1} - s) \times ({m}_{2}/{m}_{1})$ given $S=s$. Therefore, the observable survival time ${Y}_{1}$ for the participants without and with treatment switching from the active control group to the experimental group will be $\text{min}\left({T}_{1}, {C}_{1}\right)$ and $\text{min}({T}_{1}^{*}, {C}_{1})$, respectively. The observable survival time ${Y}_{2}$ for the participants in the experimental group will be $\text{min}({T}_{2}, {C}_{2})$. Finally, a non-inferiority test using DRMST between the two samples $\{{Y}_{1}\}$ and $\{{Y}_{2}\}$ in ITT analysis is performed (Fig. 3). However, using the margin $\delta$ designed for NI trials without treatment switching will inflate the type I error in NI trials with treatment switching. If ${m}_{2}/{m}_{1}<1$ with ${R}_{2}\left(\tau \right)={R}_{1}\left(\tau \right)-\delta$ (null hypothesis holds), when participants switch treatment from the active control group to the experimental group, the type I error cannot be controlled because ${R}_{2}\left(\tau \right)>{R}_{1}^{*}\left(\tau \right)-\delta$ in ITT analysis. Here, ${R}_{1}^{*}\left(\tau \right)$ (< ${R}_{1}\left(\tau \right)$) represents the RMST of the mixed active control group, which includes participants with and without treatment switching. For NI trials involving treatment switching, we propose to modify the (unadjusted) margin $\delta$ to the adjusted margin ${\delta }^{*}={R}_{1}^{*}\left(\tau \right)-{R}_{1}\left(\tau \right)+\delta$. That ensures ${R}_{1}^{*}\left(\tau \right)-{\delta }^{*}={R}_{1}\left(\tau \right)-\delta$. Thus, testing whether ${R}_{2}\left(\tau \right)>{R}_{1}^{*}\left(\tau \right)-{\delta }^{*}$ in data with switching can determine whether ${R}_{2}\left(\tau \right)>{R}_{1}\left(\tau \right)-\delta$ in data without switching. The adjusted margin ${\delta }^{*}$ does not change the interpretation of the margin $\delta$ regarding the DRMST between the (pure) active control and the experimental groups and is solely used for testing purposes. When the null hypothesis is rejected in ITT analysis, we still conclude that the RMST of the experimental group is not less than or equal to that of the (pure) active control group by $-\delta$. Notably, the adjusted margin ${\delta }^{*}$ is automatically generated when ${R}_{1}\left(\tau \right)$, $\delta$, and ${R}_{1}^{*}\left(\tau \right)$ are determined during the design stage. ${R}_{1}\left(\tau \right)$ and $\delta$ are calculated from the parameters required for power calculation, and ${R}_{1}^{*}\left(\tau \right)$ is estimated by averaging $\left\{{\widehat{R}}_{1,l}^{*}\left(\tau \right), l=1, 2,\dots \right\}$ via simulations, where ${\widehat{R}}_{1,l}^{*}\left(\tau \right)$ is the RMST estimate of the mixed active control group, calculated by the area under the KM curve using $\{{Y}_{1}\}$ from the $l$-th simulated data.

### The proposed *nifts*

The proposed *nifts* offers three options for a non-inferiority margin, assumes three entry patterns and generalized gamma distributions for event time, incorporates two distributions for dropout censoring, and provides five distribution options for switching time. Power and sample size calculation is implemented through two main functions: *calculate_power* and *calculate_size*. The first function calculates power and outputs the associated expected number of events in the active control and experimental groups. The latter determines the required sample size to achieve a specified power with the adjusted margin. The required sample size is obtained by a monotonically increasing power curve to the sample sizes. This curve is estimated using a monotonic smoothing technique [18] based on a finite number of power points and sample sizes.

The *calculate_power* function includes 25 parameters to simulate various scenarios: $n$*, *$r$*, *${m}_{1}$*, *${m}_{2}$*, shape, k, *${f}_{1}$*, *${m}_{0}$*, *${f}_{2}$*, margin, *${p}_{s}$*, *${r}_{s}$*, *${\rho }_{s}$*, s.dist, entry, censoring.prob, lossfu.dist,*${T}_{a}$*, *${T}_{e}$*, *$\tau$*, one.sided.alpha, TXswitch, af, n_simulations,* and *seed*. Detailed descriptions for these parameters are provided in Table 2. When ${f}_{1}$ is provided, the first margin option is used. When ${f}_{2}$ and ${m}_{0}$ are provided, the second margin option is used. When a numeric *margin* is provided, a customized margin is applied, for example, an RMST margin converted from an HR margin.

**Table 2:** Descriptions for parameters in *nifts* package

*calculate_power*		Inputs
*n*	Sample size of the active control group	Integer
*r*	Allocation ratio: the ratio of the participants in the experimental group to those in the active control group	Default = 1
*m*_1_	Median survival time in the active control group	Numeric value
*m*_2_	Median survival time in the experimental group	Numeric value
*Shape*	The shape parameter in generalized gamma distributions	Default = 1
*k*	The $k$ parameter in generalized gamma distributions	Default = 1
*f*_1_	Preserved fraction (0 <${f}_{1}$ < 1)	If (no *margin*) { input either${f}_{1}$or (${m}_{0}$,${f}_{2}$) } else *margin*
*m*_0_	Median survival time in the hypothetical placebo group	
*f*_2_	Preserved fraction (0 <${f}_{2}$ < 1)	
*margin*	A non-inferiority margin (> 0) for DRMST	
*p*_s_	Switching probability	Default = 0.2
*r*_s_	Ratio of$E(S)$ to $E({T}_{1})$	Default = 0.5
*ρ*_s_	Correlation of $S$ and ${T}_{1}$	Default = 0.775
*s.dist*	Options for the switching time distribution (*s.dist* = “unif”, “beta”, “gamma”, “indepExp”, or = a numeric value)	Default = “gamma”
*entry*	Entry patterns: “increasing”, “decreasing”, or “unif”	Default = “unif”
*censoring.prob*	Censoring probability of the active control group under no treatment switching (*censoring.prob* = “AC.only” meaning administrative censoring only, or = a numeric value)	Default = “AC.only”
*lossfu.dist*	Options for the dropout censoring distribution: uniform (“unif”) or exponential (“exp”)	Default = “unif”
*T*_a_	Accrual duration	Numeric value
*T*_e_	Trial duration	Numeric value
*τ*	A prespecified time for RMST calculation	Default = ${T}_{e}$
*one.sided.alpha*	One-sided significance level	Default = 0.025
*TXswitch*	Direction of treatment switching (*TXswitch* = “1to2” or “2to1”)	Default = “1to2”
*af*	A multiplier for the accelerated factor	Default = 1
*n_simulations*	Number of simulations	Default = 5000
*seed*	Simulation seed for reproducibility	Default = 2024
***calculate_size*** (includes the following four parameters and the same parameters as *calculate_power*, except for)		
*n*_*L*_	Minimum sample size	Integer
*n*_U_	Maximum sample size	Integer
*B*	Number of intervals splitting upper and lower bound	Default = 10
*epwr*	An expected power	Default = 0.80

The *calculate_size* function uses the same parameters as *calculate_power,* except for* n*, and adds 4 parameters: ${n}_{L}$*, *${n}_{U}$*, *$B$*, epwr*. The lower (${n}_{L}$) and upper (${n}_{U}$) bounds are minimum and maximum sample sizes users input when exploring sample sizes for an expected power (*epwr*). The function divides the range of the bounds into $B$ equal intervals and calculate the powers at $n$ = ${n}_{L}$ + $j\times w$, where $w$ = round(${(n}_{U}{ - n}_{L})/B$) and $j$ = 0, 1, 2, …, $B$. A shape constrained additive model [18] is employed to fit a monotonically increasing power curve to the sample sizes, from which the required sample size is determined.

## Results

### Parameters setting via real-world examples

The first example is an open-label phase III trial comparing survival benefits in patients with chemotherapy-refractory metastatic colorectal cancer, who were randomly assigned to either panitumumab + best supportive care (BSC) or BSC alone [13]. A total of 231 patients were randomly assigned to panitumumab + BSC, and 232 to BSC alone. Among the BSC alone patients, 85% experienced disease progression, and 76% switched to panitumumab + BSC after evaluation by the investigator. Thus, the switch probability ${p}_{s}$ was 0.89 (= 0.76/0.85). We use the trial scenario (ClinicalTrial.gov: NCT00113763) to illustrate the proposed method for power and sample size calculation in NI trials with treatment switching when using DRMST in ITT analysis. We set ${T}_{a}$ = 0, ${T}_{e}$ = 26 (in months), $n$ = 232 with a 1:1 allocation ratio ($r$= 1), and a censoring probability of 0.05 for BSC alone group. We compare the RMSTs of overall survival at $\tau$ = 12 and 18 (in months), which approximately correspond to the minimum and median follow-up times (52 and 72 weeks), between the two groups with a preserved fraction of ${f}_{1}$ = 0.8 (i.e., margin = $0.2{R}_{1}(\tau )$). We assume Weibull distributions with ${m}_{1}$ = 6.0 and ${m}_{2}$ = 6.4 and a shape parameter of 1 for event time. If there were no treatment switching, the power at $n=$ 232 and 306 could reach 90% at a one-sided significance level of 0.005 for $\tau$ = 12 and 18, respectively.

Next, we examine the changes in powers and required sample sizes when treatment switching occurs with a switch probability of ${p}_{s}$ = 0.89. We assume *s.dist* = “gamma” or “indepExp” with ${r}_{s}$ = 0.3 (= 1.96/6.4, the ratio of $E(S)$ = 1.96 (the reported mean PFS) to $E({T}_{1})$ = 6.4). For *s.dist* = “gamma”, we assume ${\rho }_{s}$ = 0.1, 0.3, 0.5, 0.7, and 0.9 to model low to high correlations between progression-free survival and overall survival. For $\tau$ = 12, the resulting powers at $n=$ 232 with adjusted margins are close to 0.9. The required sample sizes to achieve a power of 0.9 with adjusted margins range from 232 to 240 (Table 3). For $\tau$ = 18, the resulting powers at $n=$ 306 with adjusted margins are close to 0.9. The required sample sizes to achieve a power of 0.9 with adjusted margins range from 310 to 313 (Table 3).

**Table 3:** Powers and required sample sizes with adjusted margins in NI trials allowing treatment switching with a switch probability of *p*_s_ = 0.89, when using DRMST in ITT analysis

τ = 12	*s.dist* = “gamma”					*s.dist* = “indepExp”
*ρ*_s_	0.1	0.3	0.5	0.7	0.9	0
Power at a one-sided significance level of 0.005 with *n* = 306 and *r* = 1	0.898	0.894	0.898	0.900	0.887	0.891
Required sample sizes (*n*) to achieve the power of 0.9 at the one-sided significance level of 0.005	237	240	236	232	237	237
τ = 18	*s.dist* = “gamma”					*s.dist* = “indepExp”
*ρ*_s_	0.1	0.3	0.5	0.7	0.9	0
Power at a one-sided significance level of 0.005 with *n* = 232 and *r* = 1	0.892	0.889	0.894	0.895	0.895	0.891
Required sample sizes (*n*) to achieve the power of 0.9 at the one-sided significance level of 0.005	313	313	311	312	310	313

The second example is a non-inferiority trial involving 1,234 women with early-stage breast cancer who have undergone breast-conserving surgery [15]. This trial compares hypofractionated radiotherapy to standard radiotherapy for preventing local recurrence of invasive breast cancer. Between April 1993 and September 1996, 622 and 612 patients were randomly assigned to hypofractionated radiotherapy and standard radiotherapy, respectively, and were followed up to 12 years (${T}_{a}$ = 3.5, ${T}_{e}$ = 12, and $r$ = 1) with 7.9% dropout censoring. Among the patients randomized to hypofractionated radiotherapy, 1.2% selected standard radiotherapy instead (${p}_{s}$ = 0.012, $S$ = 0, and *TXswitch* = “2to1”) [9]. Given the assumption of a 7% 5-year local recurrence rate for standard radiotherapy [15], we assume Weibull distributions with ${m}_{1}$ = ${m}_{2}$ = $-$ 5 log(2)/log(0.93) = 47.8 and a shape parameter of 1 for event time. Moreover, we assume a dropout censoring probability of 0.04 (about a half of 0.079) for standard radiotherapy. Together with an administrative censoring probability of 0.862, the overall censoring probability for standard radiotherapy is 0.902, i.e., *censoring.prob* = 0.902. Based on the hypofractionated radiotherapy is not worse than the standard radiotherapy by 5% in local recurrence-free survival at 5 years, the HR margin is $1/\theta$ = log(0.88)/log(0.93) = 1.762.

We compare the RMSTs at $\tau$ = 5.75 and 10 (corresponding to two analysis times in [15]) between the two radiotherapy groups. The DRMST margins, converted from the HR margin, are 0.169 and 0.484, respectively. With a one-sided significance level of 0.05 and a power of 0.9, the required sample sizes $n$ with adjusted margins in the standard radiotherapy group are 560 for $\tau$ = 5.75 and 376 for $\tau$ = 10, compared to 558 and 374 without treatment switching. The results are very similar with and without switching since the switching probability of 0.012 is small.

### Simulation scenarios

Various simulations are conducted to examine the impact of treatment switching on power and sample size estimation in NI trials using DRMST in ITT analysis. These simulations consider different relative effectiveness of the experimental versus the active control group (${m}_{2}/{m}_{1}$, ${m}_{1}$ = 1), switching times (${r}_{s}$ = 0.5 and 0.25), switching probabilities (${p}_{s}$ = 0.2 and 0.4), event time distributions (Weibull distributions with *shape* = 1, 0.75, and 1.25), entry patterns (“decreasing”, “uniform”, “increasing”), dropout censoring distributions (“uniform”, “exponential”), switching time distributions (*s.dist* = “unif”, “beta”, “gamma”, and “indepExp”), and allocation ratios ($r$ = 1 and 2). For each scenario, we set ${T}_{a}$ = 3, ${T}_{e}$ = 5, $\tau$= 5, ${\rho }_{s}$ = 0.775, *censoring.prob* = 0.2, and *n_simulations* = 5000, unless otherwise specified. The one-sided significance level is set at 0.025.

#### Performance on type I errors

We first examine the performance of type I errors using adjusted margins when treatment switching is present. We set ${m}_{2}$ to 0.9 and 0.8 and determine $\delta$ such that ${R}_{2}\left(\tau \right)={R}_{1}\left(\tau \right)-\delta$. Three entry patterns and two dropout censoring distributions are considered. We present results for ${r}_{s}$ = 0.5, *shape* = 1, *s.dist* = “gamma”, and $r$ = 1, since the results for the other parameter setting are similar. The type I errors with unadjusted margins $\delta$ are inflated, while those with adjusted margins ${\delta }^{*}$ are well-controlled, except in the scenario of “increasing” entry (Table 4). The type I errors using the adjusted margins ${\delta }^{*}$ exhibit a slight inflation of up to 0.005 compared to the pre-specified type I error of 0.025. This inflation occurs because most participants in the “increasing” entry scenario have follow-up times less than 5, resulting in unsatisfactory RMST estimates at $\tau$ = 5. When $\tau$ is considered as 4 with the same simulated data, the type I errors are reduced and are very close to or below the pre-specified type I error (Supplementary Table s1).

**Table 4:** Type I errors when *R*_2_ (τ) = *R*_1_ (τ) - δ, given *n* = 628 and a one-sided significance level of 0.025

Uniform distributions for dropout censoring				
* m*_2 _= 0.9		Entry pattern		
	Margin	Decreasing	Uniform	Increasing
*p*_s_= 0.2	adjusted	.025	.025	.029
	unadjusted	.040	.036	.038
* p*_*s*_= 0.4	adjusted	.026	.026	.029
	unadjusted	.058	.054	.052
*m*_s_= 0.8				
	Margin	Decreasing	Uniform	Increasing
*p*_s_= 0.2	adjusted	.024	.023	.029
	unadjusted	.053	.051	.054
*p*_s_= 0.4	adjusted	.024	.025	.030
	unadjusted	.116	.114	.115
Exponential distributions for dropout censoring				
*m*_2_= 0.9		Entry pattern		
	Margin	Decreasing	Uniform	Increasing
*p*_s_= 0.2	adjusted	.025	.025	.028
	unadjusted	.036	.037	.035
*p*_s_= 0.4	adjusted	.025	.025	.029
	unadjusted	.052	.054	.053
*m*_2_= 0.8				
	Margin	Decreasing	Uniform	Increasing
*p*_s_= 0.2	adjusted	.022	.025	.030
	unadjusted	.052	.052	.057
*p*_s_= 0.4	adjusted	.026	.026	.029
	unadjusted	.112	.112	.110

#### Performance on power and sample sizes

We use ${m}_{0}$ = 0.5 and ${f}_{2}$ = 0.5 to determine ${R}_{0}(\tau )$ and $\delta$ = 0.5 $\left({R}_{1}\left(\tau \right)-{R}_{0}\left(\tau \right)\right)$. With adjusted margins, there is no significant change in power with ${n}_{ns}$ when ${m}_{2}$> ${m}_{1}$ (${m}_{2}/{m}_{1}$ = 1.1) and ${m}_{2}$< ${m}_{1}$ (${m}_{2}/{m}_{1}$= 0.9), even with an increase in ${p}_{s}$ from 0.2 to 0.4 and a reduction in ${r}_{s}$ from 0.5 to 0.25. The changes in power are less than 0.015. The ratios ($n/{n}_{ns}$) of sample sizes with treatment switching ($n$) to those without switching (${n}_{ns}$) are close to 1, ranging from 0.968 to 1.045 (Tables 5 and 6). The performance on power and sample sizes is not sensitive to the choice of switch time distributions and switching times (Supplementary Table s2).

**Table 5:** Required sample sizes (*n*) and powers at *n*_*ns*_ with *r*_*s*_ = 0.5, shape = 1, and *r* = 1, where *n*_*ns*_denotes the sample size under no treatment switching, given a power of 0.8 and a one-sided significance level of 0.025

*m*_2_/*m*_1_ = 1.1 *n*_*ns*_= 157; E1 = 125.5; E2 = 121.7		*s.dist*			
		unif	beta	gamma	indepExp
*p*_s _= 0.2	*n*	154	160	154	152
	E1	122.6	127.6	122.8	121.0
	E2	119.4	124.1	119.3	117.8
	$n/n_{ns}$	0.981	1.019	0.981	0.968
	power at $n_{ns}$	0.803	0.802	0.800	0.797
	Power at *n*	0.792	0.805	0.790	0.783
*p*_s_ = 0.4	*n*	158	160	162	162
	E1	125.6	127.1	130.3	128.4
	E2	122.6	124.1	127.2	125.6
	$n/n_{ns}$	1.006	1.019	1.032	1.032
	Power at $n_{ns}$	0.803	0.806	0.802	0.792
	Power at *n*	0.798	0.809	0.811	0.806
*m*_2_/*m*_1_ = 0.9 *n*_*ns*_ = 643; E1 = 514.3; E2 = 529.9		*s.dist*			
		unif	beta	gamma	indepExp
*p*_s _= 0.2	*n*	633	637	630	639
	E1	507.7	510.7	505.5	513.0
	E2	521.8	525.2	519.5	526.7
	$n/n_{ns}$	0.984	0.991	0.980	0.994
	Power at$n_{ns}$	0.802	0.803	0.793	0.812
	Power at *n*	0.796	0.794	0.789	0.802
*p*_s _= 0.4	*n*	641	638	628	635
	E1	515.5	513.4	505.0	511.9
	E2	528.4	526.0	517.9	523.8
	$n/n_{ns}$	0.997	0.992	0.977	0.988
	Power at $n_{ns}$	0.804	0.799	0.808	0.810
	Power at *n*	0.808	0.801	0.797	0.814

E1 and E2 are the expected number of events in the active control and experimental groups

**Table 6:** Required sample sizes (*n*) and powers at *n*_*ns*_ with *r*_*s*_ = 0.25, *shape* = 1 and *r* = 1, where *n*_*ns*_ denotes the sample size under no treatment switching, given a power of 0.8 and a one-sided significance level of 0.025

*m*_2_/*m*_1_ = 1.1 *n*_*ns*_= 157; E1 = 125.5; E2 = 121.7		*s.dist*			
		unif	beta	gamma	indepExp
*p*_s _= 0.2	*n*	-	157	155	152
	E1	-	124.9	123.4	120.9
	E2	-	121.8	120.1	117.8
	$n/n_{ns}$	-	1.000	0.987	0.968
	Power at ${n}_{ns}$	-	0.801	0.802	0.796
	Power at *n*	-	0.801	0.798	0.783
*p*_s _= 0.4	*n*	-	156	163	164
	E1	-	123.6	129.0	129.7
	E2	-	120.9	126.5	127.0
	$n/{n}_{ns}$	-	0.994	1.038	1.045
	Power at ${n}_{ns}$	-	0.802	0.802	0.791
	Power at *n*	-	0.794	0.814	0.805
*m*_2_/*m*_1_ = 0.9 ${n}_{ns}$ = 643; E1 = 514.3; E2 = 529.9		*s.dist*			
		unif	beta	gamma	indepExp
*p*_s_ = 0.2	*n*	-	648	626	640
	E1	-	520.4	503.1	514.2
	E2	-	534.1	516.1	527.6
	$n/{n}_{ns}$	-	1.008	0.974	0.995
	Power at *n*_*ns*_	-	0.800	0.795	0.811
	Power at *n*	-	0.801	0.794	0.798
*p*_s _= 0.4	*n*	-	629	628	628
	E1	-	507.5	506.5	507.1
	E2	-	518.7	517.9	518.0
	$n/{n}_{ns}$	-	0.978	0.977	0.977
	Power at *n*_*ns*_	-	0.809	0.811	0.813
	Power at *n*	-	0.807	0.802	0.795

E1 and E2 are the expected number of events in the active control and experimental groups

- *s.dist* = “unif” does not satisfy *r*_*s*_= 0.25

We also change the shape parameter in Weibull distributions to evaluate the impact of different event time distributions (Supplementary Figure s1). Similarly, with adjusted margins, there is no significant change in power with ${n}_{ns}$. The power changes are less than 0.026. The ratios of $n/{n}_{ns}$ remain close to 1, ranging from 0.934 to 1.013 (Supplementary Tables s3 and s4). When the shape value decreases, the required sample size increases.

Moreover, we evaluate the impact of allocation ratio and entry patterns on power and sample size calculation. When we change the allocation ratio from 1 to 2, the power change and size ratios are similar, but more total sample sizes ($n(r+1)$) are needed (Supplementary Table s5). Similarly, the entry patterns do not affect the power change and size ratios, but more sample sizes are needed in the increasing pattern (Supplementary Table s6).

We compare sample sizes assuming Weibull and gamma survival distributions. The shape and scale parameters of Weibull distribution for the active control group are 1.25 and 1.34, and the shape and scale parameters of gamma distribution are 1.53 and 0.83. These correspond to the same median survival of $m_1=1$ and a survival probability of 0.113 at *t* = 2.5. For the experimental group, we assume the same shape parameters as the active control group but with median survivals of $m_2$ = 1.1, 1.0, or 0.9. All other parameter settings are consistent with those in Supplementary Table s3. The calculated sample sizes are similar between the Weibull and gamma distributions, given the same median survival and survival probability at *t* = 2.5 (Supplementary Table s7).

## Discussion

Intention-to-treat analysis is commonly used in the final analysis of RCTs, including NI trials, even when treatment switching occurs. However, treatment switching can confound the results of ITT analysis, leading to underestimation or overestimation of treatment effects and potentially incorrect conclusions. In NI trials with treatment switching, using margins $\delta$ designed for NI trials without treatment switching can inflate type I errors in ITT analysis. Therefore, we propose modifying the margins $\delta$ to adjusted margins ${\delta }^{*}$. The type I errors with the adjusted margins ${\delta }^{*}$ are well-controlled. The adjusted margins ${\delta }^{*}$ do not change the interpretation of the margins $\delta$ regarding the DRMST between the active control and the experimental groups and are solely used for testing purposes. When non-inferiority holds in ITT analysis, we still conclude that the RMST of the experimental group is not significantly less than or equal to that of the active control group by $-\delta$. With the adjusted margins, the ratios of sample sizes with treatment switching to those without switching are close to 1, indicating no significant change in power at sample sizes calculated from the scenarios without switching. The performance on power and sample sizes is not sensitive to the choice of switch time distributions. To accelerate the computation of sample sizes, we employ a monotonic smoothing technique [18] to model the power trend as sample sizes increase. The powers at the sample size estimated by the power curve exhibit a bias of less than 2% from the expected power.

*nifts* assumes the effects of the experimental treatment are the same (common treatment effect, made by RPSFTM [21]) for participants initially in the experimental group and those who switch treatment from the active control group to the experimental group. This assumption may be problematic, as participants who switch treatment from the active control group to the experimental group may have worse survival outcomes. Properly adjusting the accelerated factor ${m}_{2}/{m}_{1}$ could help fit the scenario. Multiplying ${m}_{2}/{m}_{1}$ by a constant less than 1 might be a solution, but determining this constant value before clinical trials is challenging, even with information from previous similar studies. Nevertheless, *nifts* provides a multiplier (*af*) for the accelerated factor, enabling users to conduct sensitivity analyses if desired.

*nifts* assumes generalized gamma distributions for event times. This distribution family can accommodate most real-world scenarios, but determining the three parameters of generalized gamma distributions can be challenging for investigators. We recommend considering two-parameter gamma (setting $b$ = 1) or Weibull (setting $k$ = 1) distributions. The scale and shape parameters can be easily determined by a given median survival and a survival probability at a specific time. Their performance on sample sizes is similar, given the same median survival and survival probability at a specific time (Supplementary Table s7). In addition, *nifts* offers three entry patterns and two dropout censoring distributions for use. If needed, users can replace entry and dropout distributions in our open-source R code with more flexible distributions.

This study focuses on power and sample size calculation for NI trials with treatment switching. In the absence of treatment switching, the method by Weir and Trinquart [9] is a special case of our model. In the presence of treatment switching, the approach by Phadnis and Mayo [10] cannot be applied since the PT assumption no longer holds. The non-inferiority logrank test may be an alternative, but its margin expressed on the hazard ration scale may be problematic due to the violation of the PH assumption. Revising the margin might not be as straightforward as our approach.

## Conclusions

We propose a simulation-based approach, *nifts,* for power and sample size calculation in NI trials with treatment switching, when comparing the RMSTs of two treatment groups in ITT analysis. With its comprehensive parameter settings, *nifts* will be useful for designing NI trials that allow for treatment switching.

## Supplementary Information

Supplementary Material 1.

## Data Availability

Additional file 1: Supplementary Material. nifts is freely available at https://github.com/cyhsuTN/nifts.

## Abbreviations

HR: Hazard ratio
PH: Proportional hazards
PT: Proportion time
ITT: Intention-to-treat
RCT: Randomized controlled trials
NI: Non-inferiority
RMST: Restricted mean survival time
DRMST: Difference in restricted mean survival times
KM: Kaplan-Meier
RPSFTM: Rank preserving structural accelerated failure time models
