# Supplementary material for: Power and sample size calculation for non-inferiority trials with treatment switching in intention-to-treat analysis comparing RMSTs
Source: BMC Med Res Methodol. 2025 Jun 7;25:157. doi: 10.1186/s12874-025-02604-3 (PMC12144734; doi:10.1186/s12874-025-02604-3)
Supplement: Supplementary file 1 — Supplementary Material 1. [file 12874_2025_2604_MOESM1_ESM.docx]

**Supplemental Online Content**

**Power and sample size calculation for non-inferiority trials with treatment switching in intention-to-treat analysis comparing RMSTs**

Austin Shih^1,#^, Chih-Yuan Hsu^2,3,#,*^, and Yu Shyr^2,3,*^

^1^ Department of Mathematics, Vanderbilt University, Nashville, TN 37240, United States

^2^ Department of Biostatistics, Vanderbilt University Medical Center, Nashville, TN 37203, United States

^3^ Center for Quantitative Sciences, Vanderbilt University Medical Center, Nashville, TN 37203, United States

^#^Austin Shih and Chih-Yuan Hsu contributed equally to this work

^*^Correspondence: Chih-Yuan Hsu: [chih-yuan.hsu@vumc.org](mailto:chih-yuan.hsu@vumc.org); Yu Shyr: [yu.shyr@vumc.org](mailto:yu.shyr@vumc.org)

- **Details for censoring distribution**
- **Parameters determination in beta, gamma, uniform, and independent exponential distributions via** $\boldsymbol{r}_{\boldsymbol{s}}$ **and** $\boldsymbol{\rho}_{\boldsymbol{s}}$
- **Figure s1.** Weibull survival functions with different shape values, $m_{1}$ = 1, and $m_{2}$ = 1.1 (left) and 0.9 (right).
- **Table s1.** Type I errors for “increasing” entry when $R_{2}\left( \tau\right)=R_{1}\left( \tau\right)-\delta$ at $\tau$ = 4.
- **Table s2.** Required sample sizes ($n$) and powers at $n_{ns}$ with $r_{s}$ = 0.5 and 0.25, *shape* = 1 and $r$ = 1, under $m_{2}/m_{1}$ = 1.
- **Table s3.** Required sample sizes ($n$) and powers at $n_{ns}$ with $r_{s}$ = 0.5, *shape* = 1.25, and $r$ = 1.
- **Table s4.** Required sample sizes ($n$) and powers at $n_{ns}$ with $r_{s}$ = 0.5, *shape* = 0.75, and $r$ = 1.
- **Table s5.** Required sample sizes ($n$) and powers at $n_{ns}$ with $r_{s}$ = 0.5, *shape* = 1, and $r$= 2.
- **Table s6.** Required sample sizes ($n$) and powers at $n_{ns}$ with $r_{s}$ = 0.5, shape = 1, and $r$ = 1, under various entry patterns.
- **Table s7.** Comparison between Weibull and gamma survival distributions in sample sizes required to achieve a power of 0.8.

**Details for censoring distribution**

The censoring consists of both dropout censoring and administrative censoring. The distribution of the censoring can be expressed as follows:

$$f\left( c | v \right)=d\left( c \right)I\left( 0<c<T_{e}-v \right)+ \bar{D}\left( T_{e}-v \right)I\left( c=T_{e}-v \right).$$

The dropout censoring density function is assumed to be either $d(c) = h^{-1} I(0 < c < h)$ (uniform) or $d\left( c \right)=\lambda e^{-\lambda c}I(0<c)$ (exponential). Given the entry distribution $f_{V}(v)$, the value of $h$ or $\lambda$ is determined by a pre-specified censoring probability for the active control group, assuming no treatment switching. Specifically, solve $P\left( C_{1}<T_{1} \right)$ = the given censoring probability for $h$ or $\lambda$. The probability $P\left( C_{1}<T_{1} \right)$ is a function of $h$ or $\lambda$ and can be explicitly expressed as follows:

$$P\left( C_{1}<T_{1} \right)=\int_{0}^{T_{a}} P\left( C_{1}<T_{1} \right|v)f_{V}\left( v \right)dv,$$

where

$$P\left( C_{1}<T_{1} \right|v)=\int_{0}^{T_{e}-v} \int_{c}^{\infty} f\left( c | v \right)f_{T_{1}}\left( t \right)dt dc+ \int_{T_{e}-v}^{\infty} \int_{c}^{\infty} f\left( c | v \right)f_{T_{1}}\left( t \right)dt dc$$

$$=\int_{0}^{T_{e}-v} d(c)\int_{c}^{\infty} f_{T_{1}}\left( t \right)dt dc+\bar{D}\left( T_{e}-v \right)\int_{T_{e}-v}^{\infty} f_{T_{1}}\left( t \right)dt. (*)$$

If $d(c) = h^{-1} I(0 < c < h)$,

$$\left( * \right)=\int_{0}^{{min(T}_{e}-v, h)} h^{-1}\int_{c}^{\infty} f_{T_{1}}\left( t \right)dt dc+I\left( h>T_{e}-v \right)\left( 1-h^{-1}\left( T_{e}-v \right) \right)\int_{T_{e}-v}^{\infty} f_{T_{1}}\left( t \right)dt$$

$$=\int_{0}^{{min(T}_{e}-v, h)} h^{-1}S_{T_{1}}\left( c \right)dc+I\left( h>T_{e}-v \right)\left( 1-h^{-1}\left( T_{e}-v \right) \right)S_{T_{1}}\left( T_{e}-v \right).$$

For $T_{e}-T_{a}<h<T_{e}$,

$P\left( C_{1}<T_{1} \right)=\int_{0}^{T_{a}} P\left( C_{1}<T_{1} \right|v)f_{V}\left( v \right)dv$

$$=\int_{0}^{T_{e}-h} \int_{0}^{h} h^{-1}S_{T_{1}}\left( c \right)dcf_{V}\left( v \right)dv+\int_{T_{e}-h}^{T_{a}} \int_{0}^{T_{e}-v} h^{-1}S_{T_{1}}\left( c \right)dcf_{V}\left( v \right)dv$$

$$+\int_{T_{e}-h}^{T_{a}} \left( 1-h^{-1}\left( T_{e}-v \right) \right)S_{T_{1}}\left( T_{e}-v \right)f_{V}\left( v \right)dv$$

For $h\leq T_{e}-T_{a}$,

$P\left( C_{1}<T_{1} \right)=\int_{0}^{T_{a}} \int_{0}^{h} h^{-1}S_{T_{1}}\left( c \right)dcf_{V}\left( v \right)dv=\int_{0}^{h} h^{-1}S_{T_{1}}\left( c \right)dc$.

For $T_{e}\leq h$,

$$P\left( C_{1}<T_{1} \right)=\int_{0}^{T_{a}} \int_{0}^{T_{e}-v} h^{-1}S_{T_{1}}\left( c \right)dcf_{V}\left( v \right)dv+\int_{0}^{T_{a}} \left( 1-h^{-1}\left( T_{e}-v \right) \right)S_{T_{1}}\left( T_{e}-v \right)f_{V}\left( v \right)dv$$

If $d(c) = \lambda e^{-\lambda c}I(0<c)$,

$$\left( * \right)=\int_{0}^{T_{e}-v} \lambda e^{-\lambda c}S_{T_{1}}\left( c \right) dc+e^{-\lambda(T_{e}-v)}S_{T_{1}}\left( T_{e}-v \right).$$

Then,

$$P\left( C_{1}<T_{1} \right)=\int_{0}^{T_{a}} \left( \int_{0}^{T_{e}-v} \lambda e^{-\lambda c}S_{T_{1}}\left( c \right) dc+e^{-\lambda\left( T_{e}-v \right)}S_{T_{1}}\left( T_{e}-v \right) \right)f_{V}\left( v \right)dv.$$

**Parameters determination in beta, gamma, uniform, and independent exponential distributions via** $\boldsymbol{r}_{\boldsymbol{s}}$ **and** $\boldsymbol{\rho}_{\boldsymbol{s}}$

We consider using $r_{s}$ and $\rho_{s}$ to determine the parameters in the assumed distributions for the switching time, where $r_{s}=E(S)/E\left( T_{1} \right)$ denotes the ratio of the average switching time to the average survival time of the active control group, and $\rho_{s}$ denotes the correlation between $S=XT_{1}$ and $T_{1}$. When $r_{s}$ and $\rho_{s}$ are given, the parameters in the assumed distributions can be obtained through solving the two equations: $r_{s}=E(S)/E\left( T_{1} \right)=E(X)$ and

$$\rho_{s}=cor\left( S, T_{1} \right)=\frac{E\left( XT_{1}^{2} \right)-E\left( XT_{1} \right)E\left( T_{1} \right)}{\sqrt{Var\left( S \right)}\sqrt{Var\left( T_{1} \right)}}=\frac{E\left( X \right)\sqrt{Var\left( T_{1} \right)}}{\sqrt{E\left( X^{2} \right)Var\left( T_{1} \right)+Var\left( X \right)\left( E\left( T_{1} \right) \right)^{2}}}$$

$$=\frac{E\left( X \right)\sqrt{Var\left( T_{1} \right)}}{\sqrt{\left( E\left( X \right) \right)^{2}Var\left( T_{1} \right)+Var\left( X \right)E\left( T_{1}^{2} \right)}}$$

where

$$Var\left( s \right)=E\left( X^{2} \right)E\left( T_{1}^{2} \right)-\left( E\left( X \right) \right)^{2}\left( E\left( T_{1} \right) \right)^{2}$$

$$=E\left( X^{2} \right)E\left( T_{1}^{2} \right)-E\left( X^{2} \right)\left( E\left( T_{1} \right) \right)^{2}+{E\left( X^{2} \right)\left( E\left( T_{1} \right) \right)^{2}-\left( E\left( X \right) \right)}^{2}\left( E\left( T_{1} \right) \right)^{2}$$

$$=E\left( X^{2} \right)Var\left( T_{1} \right)+Var\left( X \right)\left( E\left( T_{1} \right) \right)^{2}.$$

When $S=XT_{1}$ and $X\sim\mathrm{Beta}(a,b)$, solving $r_{s}=a/(a+b)$ and $\rho_{s}=\left\{ \frac{a}{a+b}\sqrt{Var\left( T_{1} \right)} \right\}/ \left\{ \left( \frac{a}{a+b} \right)^{2}Var\left( T_{1} \right)+\frac{ab}{\left( a+b \right)^{2}\left( a+b+1 \right)}E\left( T_{1}^{2} \right) \right\}^{1/2}$ for $a$ and $b$. When $S=XT_{1}$ and $X\sim\mathrm{Gamma}(a,b)$, solving $r_{s}=a/b$ and $\rho_{s}=\left\{ \frac{a}{b}\sqrt{Var\left( T_{1} \right)} \right\}/ \left\{ \left( \frac{a}{b} \right)^{2}Var\left( T_{1} \right)+\frac{a}{b^{2}}E\left( T_{1}^{2} \right) \right\}^{1/2}$ for $a$ and $b$. When $S$ follows an exponential distribution and is independent of $T_{1}$, the mean parameter can be determined by $E\left( S \right)=r_{s} E\left( T_{1} \right).$ When $S=XT_{1}$ and $X$ follows a uniform distribution $U(0, 1)$, i.e., $S$ follows a uniform distribution $U\left( 0, T_{1} \right)$, then $r_{s}$ equals 0.5 and $\rho_{s}=\left\{ \frac{1}{2}\sqrt{Var\left( T_{1} \right)} \right\}/ \left\{ \frac{1}{4}Var\left( T_{1} \right)+\frac{1}{12}E\left( T_{1}^{2} \right) \right\}^{1/2}$ .

**
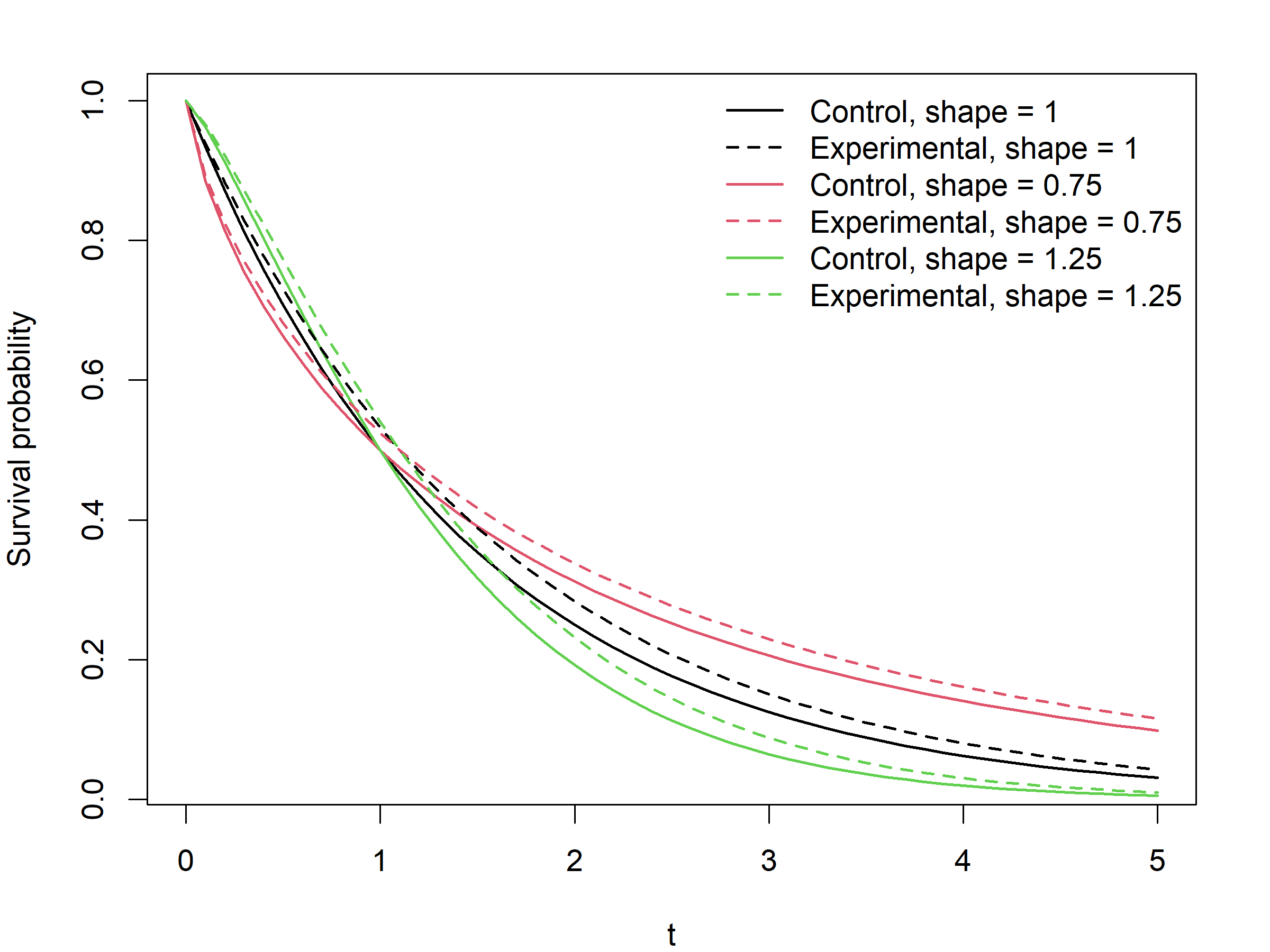

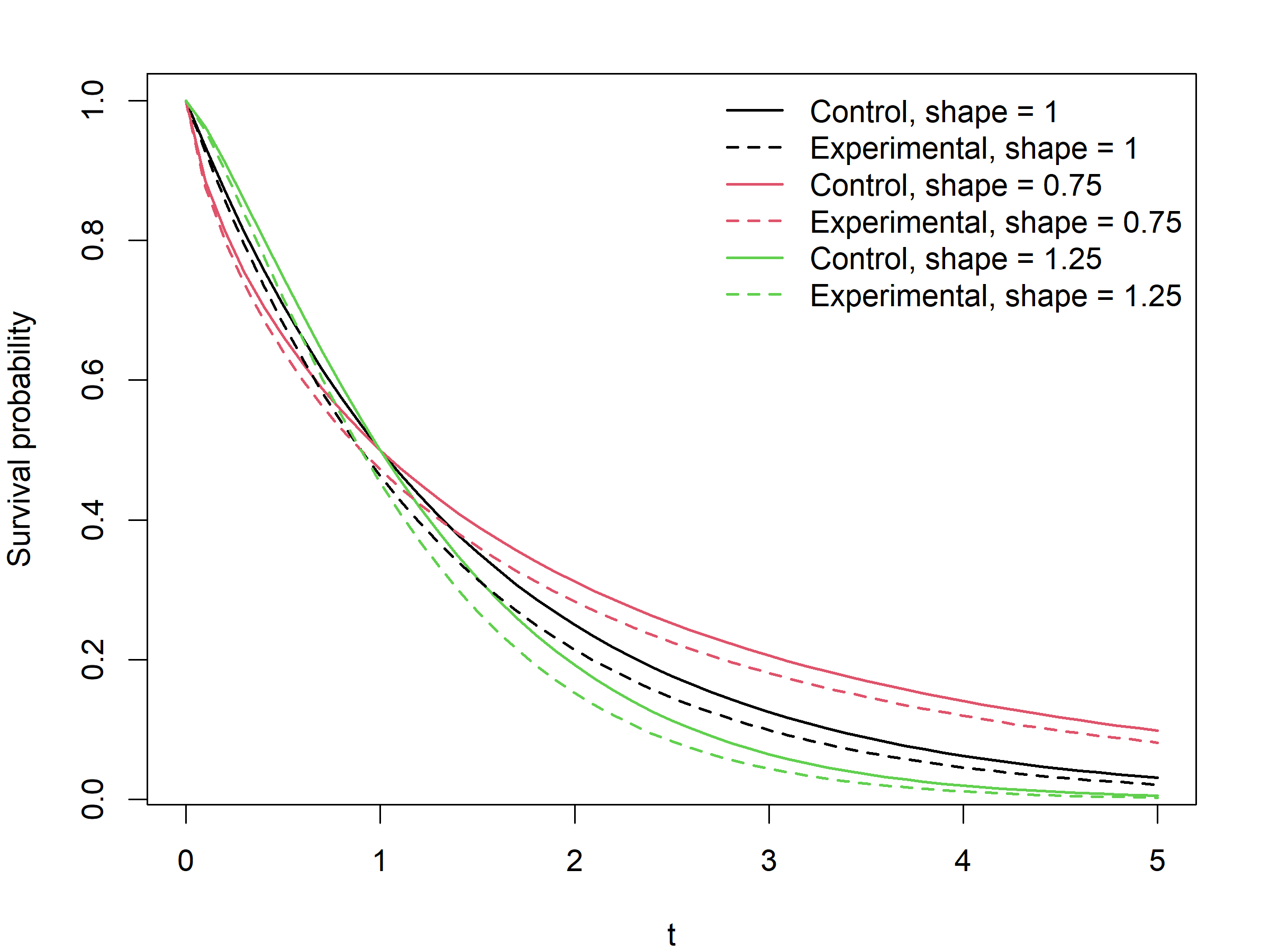
**

**Figure s1.** Weibull survival functions with different shape values, $m_{1}$ = 1 and $m_{2}$ = 1.1 (left) and $m_{1}$ = 1 and $m_{2}$ = 0.9 (right).

**Table s1.** Type I errors for “increasing” entry when $R_{2}\left( \tau\right)=R_{1}\left( \tau\right)-\delta$ at $\tau$ = 4, where $n$ = 628 and a one-sided significance level of 0.025.

|  |  | Uniform dropout censoring | | Exponential dropout censoring | |
| --- | --- | --- | --- | --- | --- |
|  |  | $m_{2}$ = 0.9 | $m_{2}$ = 0.8 | $m_{2}$ = 0.9 | $m_{2}$ = 0.8 |
| $p_{s}$ = 0.2 | adjusted | .022 | .021 | .021 | .022 |
|  | unadjusted | .034 | .052 | .034 | .052 |
| $p_{s}$ = 0.4 | adjusted | .022 | .025 | .022 | .024 |
|  | unadjusted | .048 | .115 | .054 | .108 |

**Table s2.** Required sample sizes ($n$) and powers at $n_{ns}$ with $r_{s}$ = 0.5 and 0.25, *shape* = 1 and $r$ = 1, under $m_{2}/m_{1}$ = 1. $n_{ns}$ denotes the sample size under no treatment switching, given a power of 0.8 and a one-sided significance level of 0.025. E1 and E2 are the expected number of events in the active control and experimental groups.

| $m_{2}/m_{1}$ = 1, $r_{s}$ = 0.5 $n_{ns}$ = 276; E1 = 220.7; E2 = 220.6 | | *s.dist* | | | |
| --- | --- | --- | --- | --- | --- |
|  |  | unif | beta | gamma | indepExp |
| $p_{s}$ = 0.2 | $n$ | 270 | 272 | 275 | 274 |
|  | E1 | 215.9 | 217.6 | 220.0 | 219.1 |
|  | E2 | 215.9 | 217.8 | 219.6 | 219.1 |
|  | $n/n_{ns}$ | 0.978 | 0.986 | 0.996 | 0.993 |
|  | Power at $n_{ns}$ | 0.810 | 0.801 | 0.806 | 0.802 |
|  | Power at $n$ | 0.802 | 0.805 | 0.803 | 0.809 |
| $p_{s}$ = 0.4 | $n$ | 268 | 275 | 272 | 274 |
|  | E1 | 214.4 | 220.2 | 217.7 | 219.1 |
|  | E2 | 214.2 | 220.0 | 217.7 | 219.1 |
|  | $n/n_{ns}$ | 0.971 | 0.996 | 0.986 | 0.993 |
|  | Power at $n_{ns}$ | 0.810 | 0.808 | 0.800 | 0.800 |
|  | Power at $n$ | 0.803 | 0.803 | 0.806 | 0.809 |
| $m_{2}/m_{1}$ = 1, $r_{s}$ = 0.25 $n_{ns}$ = 276; E1 = 220.7; E2 = 220.6 | | *s.dist* | | | |
|  |  | unif | beta | gamma | indepExp |
| $p_{s}$ = 0.2 | $n$ | - | 276 | 268 | 272 |
|  | E1 | - | 220.9 | 214.5 | 217.6 |
|  | E2 | - | 221.0 | 214.6 | 217.7 |
|  | $n/n_{ns}$ | - | 1.000 | 0.971 | 0.986 |
|  | Power at $n_{ns}$ | - | 0.808 | 0.806 | 0.802 |
|  | Power at $n$ | - | 0.808 | 0.800 | 0.810 |
| $p_{s}$ = 0.4 | $n$ | - | 266 | 266 | 277 |
|  | E1 | - | 212.9 | 212.8 | 221.6 |
|  | E2 | - | 212.8 | 212.8 | 221.8 |
|  | $n/n_{ns}$ | - | 0.964 | 0.964 | 1.004 |
|  | Power at $n_{ns}$ | - | 0.813 | 0.800 | 0.796 |
|  | Power at $n$ | - | 0.789 | 0.792 | 0.805 |

**Table s3.** Required sample sizes ($n$) and powers at $n_{ns}$ with $r_{s}$ = 0.5, *shape* = 1.25, and $r$ = 1. $n_{ns}$ denotes the sample size under no treatment switching, given a power of 0.8 and a one-sided significance level of 0.025. E1 and E2 are the expected number of events in the active control and experimental groups.

| $m_{2}/m_{1}$ = 1.1 $n_{ns}$ = 121; E1 = 96.7; E2 = 93.7 | | *s.dist* | | | |
| --- | --- | --- | --- | --- | --- |
|  |  | unif | beta | gamma | indepExp |
| $p_{s}$ = 0.2 | $n$ | 113 | 113 | 117 | 117 |
|  | E1 | 90.1 | 90.1 | 93.2 | 93.2 |
|  | E2 | 87.6 | 87.5 | 90.6 | 90.7 |
|  | $n/n_{ns}$ | 0.934 | 0.934 | 0.967 | 0.967 |
|  | Power at $n_{ns}$ | 0.822 | 0.815 | 0.826 | 0.825 |
|  | Power at $n$ | 0.808 | 0.804 | 0.811 | 0.813 |
| $p_{s}$ = 0.4 | $n$ | 112 | 114 | 115 | 115 |
|  | E1 | 89.0 | 90.5 | 91.4 | 91.2 |
|  | E2 | 86.7 | 88.1 | 89.1 | 89.0 |
|  | $n/n_{ns}$ | 0.926 | 0.942 | 0.950 | 0.950 |
|  | Power at $n_{ns}$ | 0.810 | 0.823 | 0.825 | 0.811 |
|  | Power at $n$ | 0.797 | 0.798 | 0.799 | 0.796 |
| $m_{2}/m_{1}$ = 0.9 $n_{ns}$ = 502; E1 = 401.4; E2 = 413.6 | | *s.dist* | | | |
|  |  | unif | beta | gamma | indepExp |
| $p_{s}$ = 0.2 | $n$ | 482 | 482 | 490 | 489 |
|  | E1 | 386.6 | 386.7 | 392.8 | 392.6 |
|  | E2 | 397.1 | 397.2 | 403.8 | 403.0 |
|  | $n/n_{ns}$ | 0.960 | 0.960 | 0.976 | 0.974 |
|  | Power at $n_{ns}$ | 0.809 | 0.813 | 0.810 | 0.818 |
|  | Power at $n$ | 0.798 | 0.802 | 0.809 | 0.807 |
| $p_{s}$ = 0.4 | $n$ | 484 | 481 | 480 | 479 |
|  | E1 | 389.7 | 386.9 | 386.6 | 386.6 |
|  | E2 | 399.0 | 396.4 | 395.7 | 395.0 |
|  | $n/n_{ns}$ | 0.964 | 0.958 | 0.956 | 0.954 |
|  | Power at $n_{ns}$ | 0.818 | 0.822 | 0.817 | 0.824 |
|  | Power at $n$ | 0.808 | 0.797 | 0.800 | 0.808 |

**Table s4.** Required sample sizes ($n$) and powers at $n_{ns}$ with $r_{s}$ = 0.5, *shape* = 0.75, and $r$ = 1. $n_{ns}$ denotes the sample size under no treatment switching, given a power of 0.8 and a one-sided significance level of 0.025. E1 and E2 are the expected number of events in the active control and experimental groups.

| $m_{2}/m_{1}$ = 1.1 $n_{ns}$ = 232; E1 = 185.5; E2 = 180.3 | | *s.dist* | | | |
| --- | --- | --- | --- | --- | --- |
|  |  | unif | beta | gamma | indepExp |
| $p_{s}$ = 0.2 | $n$ | 232 | 221 | 221 | 226 |
|  | E1 | 185.0 | 176.2 | 176.2 | 179.9 |
|  | E2 | 180.3 | 171.8 | 171.9 | 175.7 |
|  | $n/n_{ns}$ | 1.000 | 0.953 | 0.953 | 0.974 |
|  | Power at $n_{ns}$ | 0.814 | 0.812 | 0.812 | 0.812 |
|  | Power at $n$ | 0.814 | 0.800 | 0.791 | 0.802 |
| $p_{s}$ = 0.4 | $n$ | 227 | 228 | 235 | 225 |
|  | E1 | 180.4 | 181.3 | 186.8 | 178.5 |
|  | E2 | 176.6 | 177.3 | 182.7 | 175.1 |
|  | $n/n_{ns}$ | 0.978 | 0.983 | 1.013 | 0.970 |
|  | Power at $n_{ns}$ | 0.816 | 0.804 | 0.814 | 0.804 |
|  | Power at $n$ | 0.803 | 0.805 | 0.819 | 0.802 |
| $m_{2}/m_{1}$ = 0.9 $n_{ns}$ = 849; E1 = 678.8; E2 = 698.5 | | *s.dist* | | | |
|  |  | unif | beta | gamma | indepExp |
| $p_{s}$ = 0.2 | $n$ | 823 | 822 | 839 | 825 |
|  | E1 | 660.2 | 659.4 | 673.0 | 662.5 |
|  | E2 | 677.2 | 676.1 | 690.6 | 678.9 |
|  | $n/n_{ns}$ | 0.969 | 0.968 | 0.988 | 0.972 |
|  | Power at $n_{ns}$ | 0.801 | 0.811 | 0.804 | 0.811 |
|  | Power at $n$ | 0.802 | 0.802 | 0.811 | 0.804 |
| $p_{s}$ = 0.4 | $n$ | 825 | 831 | 825 | 830 |
|  | E1 | 663.5 | 668.3 | 663.9 | 669.3 |
|  | E2 | 679.1 | 683.9 | 678.8 | 683.2 |
|  | $n/n_{ns}$ | 0.972 | 0.979 | 0.972 | 0.978 |
|  | Power at $n_{ns}$ | 0.809 | 0.819 | 0.801 | 0.815 |
|  | Power at $n$ | 0.799 | 0.803 | 0.802 | 0.799 |

**Table s5.** Required sample sizes ($n$) and powers at $n_{ns}$ with $r_{s}$ = 0.5, *shape* = 1, and $r$= 2. $n_{ns}$ denotes the sample size under no treatment switching, given a power of 0.8 and a one-sided significance level of 0.025. E1 and E2 are the expected number of events in the active control and experimental groups.

| $m_{2}/m_{1}$ = 1.1 $n_{ns}$ = 124; E1 = 99.2; E2 = 192.4 | | *s.dist* | | | |
| --- | --- | --- | --- | --- | --- |
|  |  | unif | beta | gamma | indepExp |
| $p_{s}$ = 0.2 | $n$ | 122 | 121 | 122 | 119 |
|  | E1 | 97.2 | 96.4 | 97.2 | 94.8 |
|  | E2 | 189.2 | 187.8 | 189.2 | 184.5 |
|  | $n/n_{ns}$ | 0.984 | 0.976 | 0.984 | 0.960 |
|  | Power at $n_{ns}$ | 0.810 | 0.813 | 0.820 | 0.803 |
|  | Power at $n$ | 0.809 | 0.804 | 0.801 | 0.794 |
| $p_{s}$ = 0.4 | $n$ | 120 | 124 | 126 | 123 |
|  | E1 | 95.4 | 98.4 | 100.1 | 97.6 |
|  | E2 | 186.2 | 192.2 | 195.5 | 190.8 |
|  | $n/n_{ns}$ | 0.968 | 1.000 | 1.016 | 0.992 |
|  | Power at $n_{ns}$ | 0.807 | 0.813 | 0.801 | 0.799 |
|  | Power at $n$ | 0.798 | 0.813 | 0.815 | 0.802 |
| $m_{2}/m_{1}$ = 0.9 $n_{ns}$ = 502; E1 = 401.4; E2 = 827.7 | | *s.dist* | | | |
|  |  | unif | beta | gamma | indepExp |
| $p_{s}$ = 0.2 | $n$ | 500 | 511 | 495 | 497 |
|  | E1 | 401.0 | 409.9 | 397.2 | 399.2 |
|  | E2 | 824.6 | 842.7 | 816.2 | 819.5 |
|  | $n/n_{ns}$ | 0.996 | 1.018 | 0.986 | 0.990 |
|  | Power at $n_{ns}$ | 0.806 | 0.802 | 0.798 | 0.808 |
|  | Power at $n$ | 0.801 | 0.804 | 0.794 | 0.796 |
| $p_{s}$ = 0.4 | $n$ | 491 | 498 | 499 | 494 |
|  | E1 | 395.2 | 400.7 | 401.5 | 398.6 |
|  | E2 | 809.9 | 821.2 | 822.7 | 814.6 |
|  | $n/n_{ns}$ | 0.978 | 0.992 | 0.994 | 0.984 |
|  | Power at $n_{ns}$ | 0.804 | 0.803 | 0.809 | 0.807 |
|  | Power at $n$ | 0.795 | 0.805 | 0.798 | 0.808 |

**Table s6.** Required sample sizes ($n$) and powers at $n_{ns}$ with $r_{s}$ = 0.5, shape = 1, and $r$ = 1, under various entry patterns. $n_{ns}$ denotes the sample size under no treatment switching, given a power of 0.8 and a one-sided significance level of 0.025. E1 and E2 are the expected number of events in the active control and experimental groups. *s.dist* = “gamma” is used.

| $m_{2}/m_{1}$ = 1.1 | | Entry pattern | | |
| --- | --- | --- | --- | --- |
|  |  | Decreasing | Uniform | Increasing |
| No switching | $n_{ns}$ | 157 | 160 | 172 |
|  | E1 | 125.6 | 128.0 | 137.7 |
|  | E2 | 122.0 | 124.0 | 133.0 |
| $p_{s}$ = 0.2 | $n$ | 153 | 157 | 166 |
|  | E1 | 122.0 | 125.2 | 132.3 |
|  | E2 | 118.9 | 121.7 | 128.3 |
|  | $n/n_{ns}$ | 0.975 | 0.981 | 0.965 |
|  | Power at $n_{ns}$ | 0.805 | 0.806 | 0.817 |
|  | Power at $n$ | 0.793 | 0.800 | 0.812 |
| $p_{s}$ = 0.4 | $n$ | 156 | 161 | 167 |
|  | E1 | 124.0 | 127.9 | 132.6 |
|  | E2 | 121.2 | 124.8 | 129.1 |
|  | $n/n_{ns}$ | 0.994 | 1.006 | 0.971 |
|  | Power at $n_{ns}$ | 0.801 | 0.804 | 0.821 |
|  | Power at $n$ | 0.800 | 0.800 | 0.808 |
| $m_{2}/m_{1}$ = 0.9 | | Entry pattern | | |
|  |  | Decreasing | Uniform | Increasing |
| No switching | $n_{ns}$ | 627 | 636 | 687 |
|  | E1 | 501.5 | 508.8 | 549.6 |
|  | E2 | 515.5 | 524.1 | 568.0 |
| $p_{s}$ = 0.2 | $n$ | 635 | 637 | 670 |
|  | E1 | 509.3 | 511.0 | 537.9 |
|  | E2 | 522.1 | 525.1 | 553.9 |
|  | $n/n_{ns}$ | 1.013 | 1.002 | 0.975 |
|  | Power at $n_{ns}$ | 0.789 | 0.795 | 0.801 |
|  | Power at $n$ | 0.800 | 0.801 | 0.796 |
| $p_{s}$ = 0.4 | $n$ | 608 | 623 | 676 |
|  | E1 | 488.9 | 501.1 | 544.2 |
|  | E2 | 500.1 | 513.6 | 559.0 |
|  | $n/n_{ns}$ | 0.970 | 0.980 | 0.984 |
|  | Power at $n_{ns}$ | 0.805 | 0.798 | 0.813 |
|  | Power at $n$ | 0.789 | 0.799 | 0.800 |

**Table s7.** Comparison between Weibull and gamma survival distributions in sample sizes required to achieve a power of 0.8.

| $m_{2}/m_{1}$ = 1.1 | | *s.dist* | | | |
| --- | --- | --- | --- | --- | --- |
|  | Distribution | unif | beta | gamma | indepExp |
| $p_{s}$ = 0.2 | Weibull | 113 | 113 | 117 | 117 |
|  | Gamma | 112 | 110 | 113 | 114 |
| $p_{s}$ = 0.4 | Weibull | 112 | 114 | 115 | 115 |
|  | Gamma | 112 | 114 | 113 | 119 |
| $m_{2}/m_{1}$ = 1.0 | | *s.dist* | | | |
|  | Distribution | unif | beta | gamma | indepExp |
| $p_{s}$ = 0.2 | Weibull | 205 | 200 | 201 | 197 |
|  | Gamma | 196 | 199 | 202 | 208 |
| $p_{s}$ = 0.4 | Weibull | 197 | 200 | 202 | 201 |
|  | Gamma | 200 | 201 | 205 | 198 |
| $m_{2}/m_{1}$ = 0.9 | | *s.dist* | | | |
|  | Distribution | unif | beta | gamma | indepExp |
| $p_{s}$ = 0.2 | Weibull | 482 | 482 | 490 | 489 |
|  | Gamma | 478 | 486 | 483 | 480 |
| $p_{s}$ = 0.4 | Weibull | 484 | 481 | 480 | 479 |
|  | Gamma | 473 | 485 | 479 | 478 |
